# Supplementary material for: Mesoporous Nitrogen-Doped Carbon Support from ZIF-8 for Pt Catalysts in Oxygen Reduction Reaction
Source: Nanomaterials (Basel). 2025 Jan 16;15(2):128. doi: 10.3390/nano15020128 (PMC11767951; doi:10.3390/nano15020128)
Supplement: Supplementary file 1 [file nanomaterials-15-00128-s001.zip › nanomaterials-3429712-supplementary.pdf]

Supplementary Materials

Mesoporous Nitrogen-Doped Carbon Support from ZIF-8 for Pt Catalysts in Oxygen Reduction Reaction

Sangyeup Park, Jong Gyeong Kim, Youngin Cho and Chanho Pak *

Graduate School of Energy Convergence, Institute of Integrated Technology, Gwangju Institute of Science and Technology, Gwangju 61005, Republic of Korea; rupert915@gm.gist.ac.kr (S.P.); xaso123@gm.gist.ac.kr (J.G.K.); choxx576@gm.gist.ac.kr (Y.C.)

* Correspondence: chanho.pak@gist.ac.kr; Tel.: +82-62-715-5324

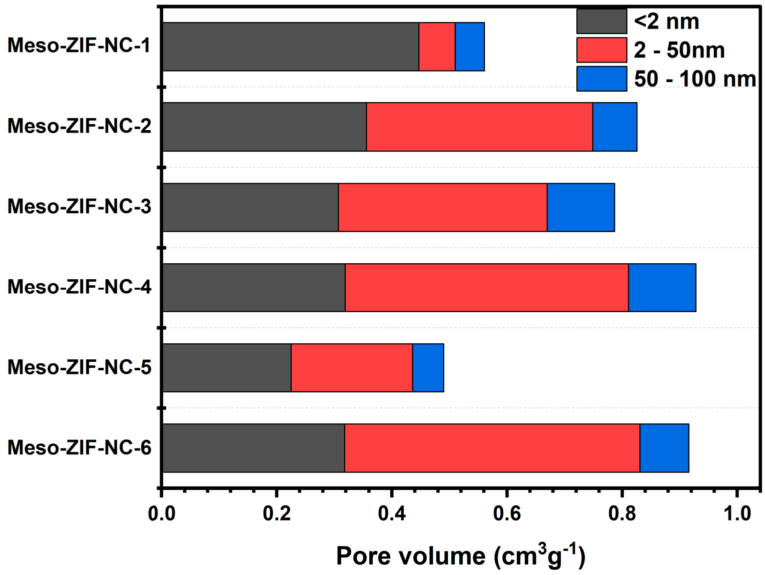

Figure S1. Pore volume graph of Meso-ZIF-NC samples.

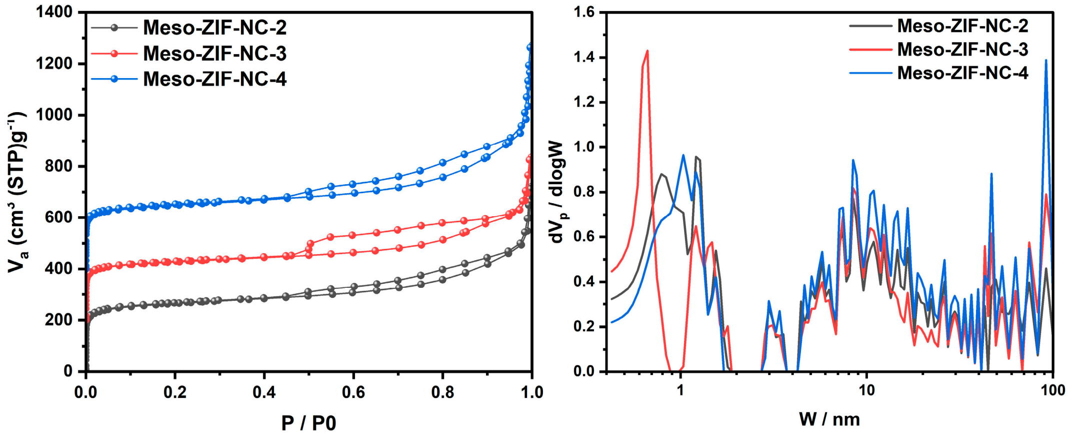

Figure S2. (a) N_2 adsorption / desorption isotherms of Meso-ZIF-NC-2,3,4 with 200 offsets and (b) pore size distribution curves of Meso-ZIF-NC-2,3,4 analyzed by NLDFT method.

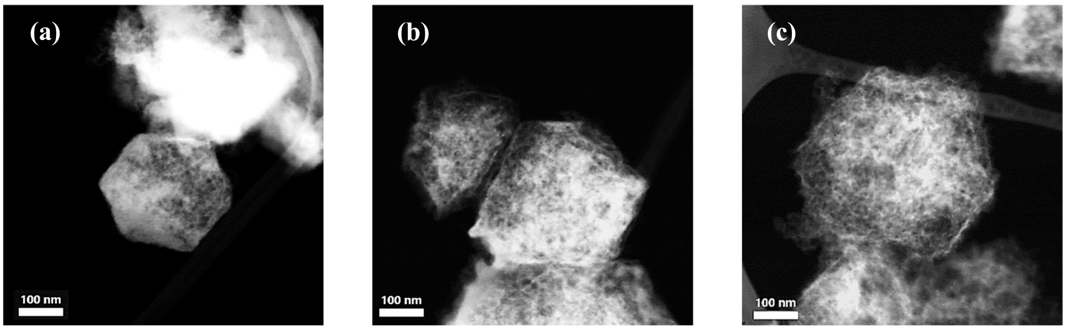

Figure S3. STEM images of (a) Meso-ZIF-NC-2, (b) Meso-ZIF-NC-3 and (c) Meso-ZIF-NC-4.

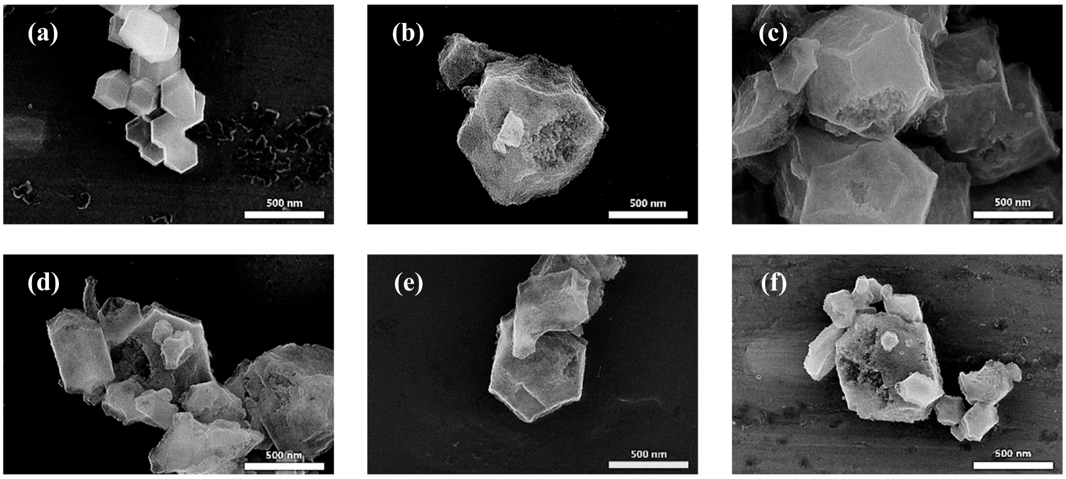

Figure S4. SEM images of (a) Meso-ZIF-NC-1, (b) Meso-ZIF-NC-2, (c) Meso-ZIF-NC-3, (d) Meso-ZIF-NC-4, (e) Meso-ZIF-NC-5, and (f) Meso-ZIF-NC-6.

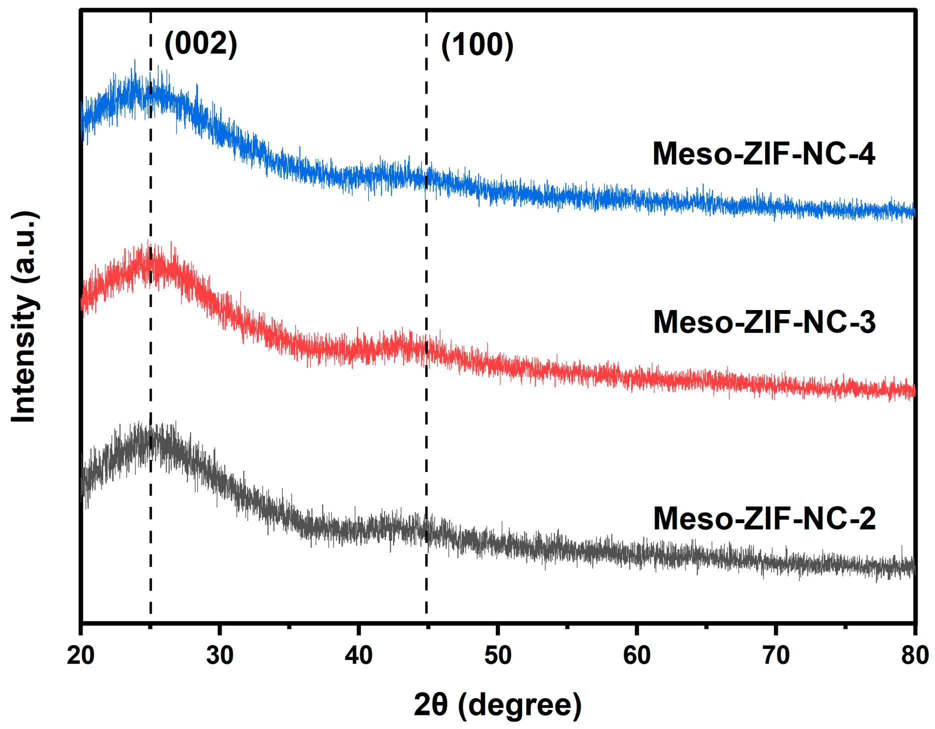

Figure S5. XRD patterns of Meso-ZIF-NC-2, 3, 4.

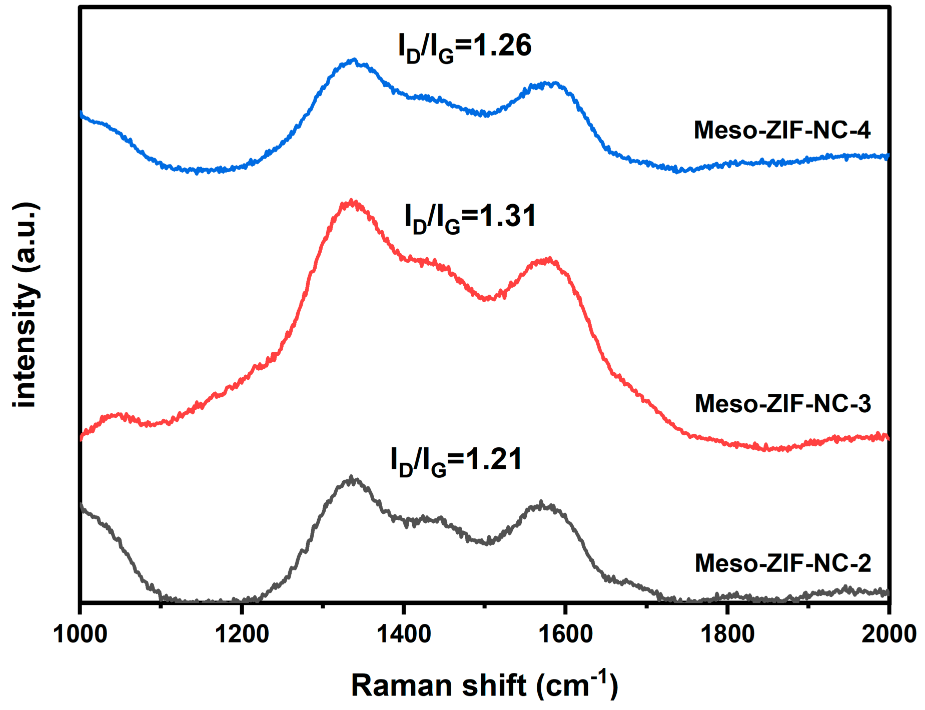

Figure S6. Raman spectra of Meso-ZIF-NC-2, 3, 4.

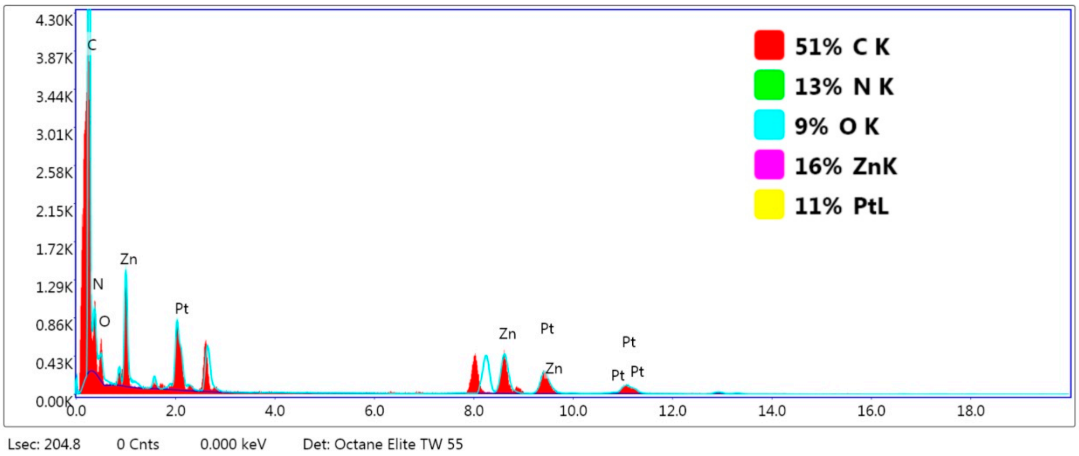

Figure S7. The sum spectrum of elemental mapping analysis for Pt/Meso-ZIF-NC-5.

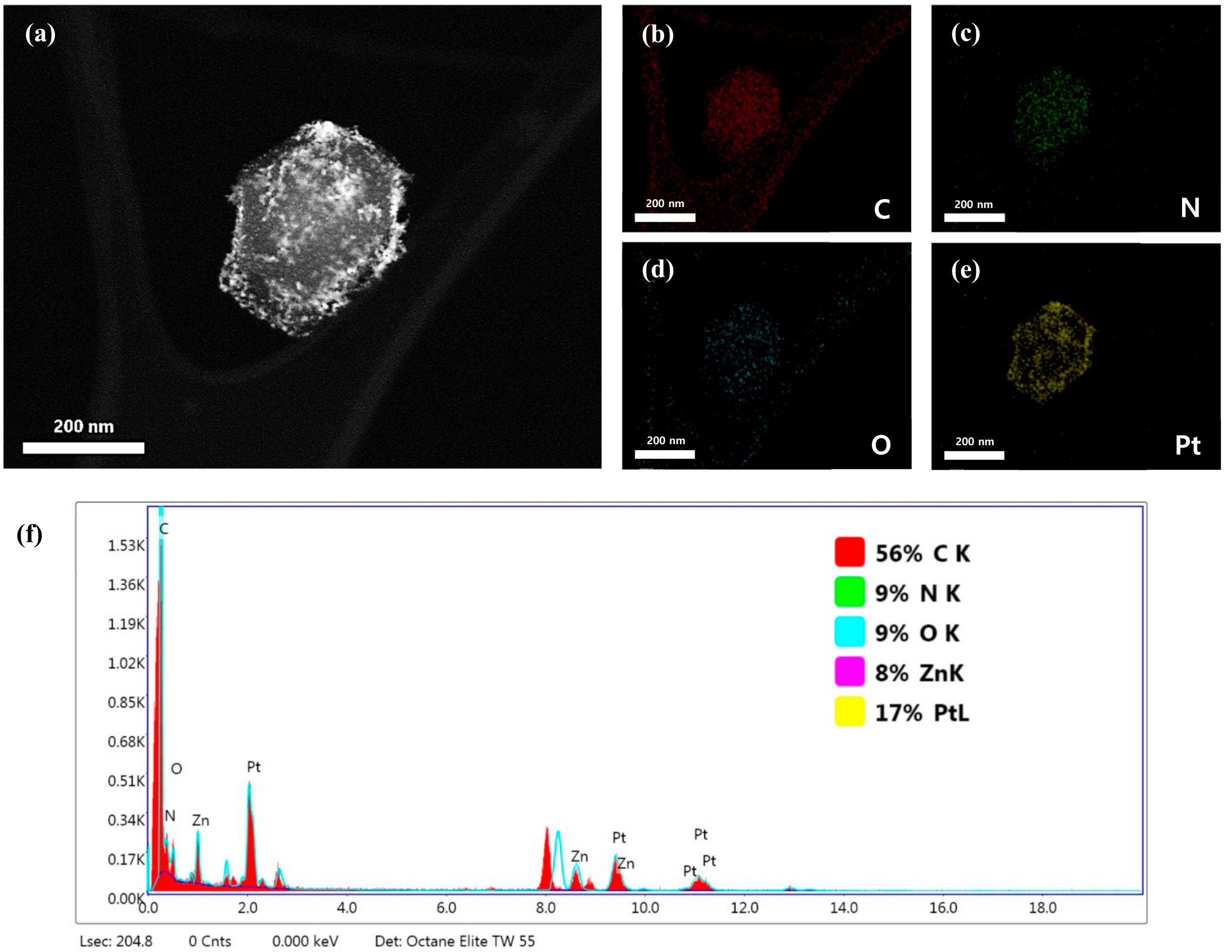

Figure S8. STEM images of (a) Pt/Meso-ZIF-NC-1, (b-e) Elemental mapping images, and (f) The sum spectrum of elemental mapping analysis for Pt/Meso-ZIF-NC-1.

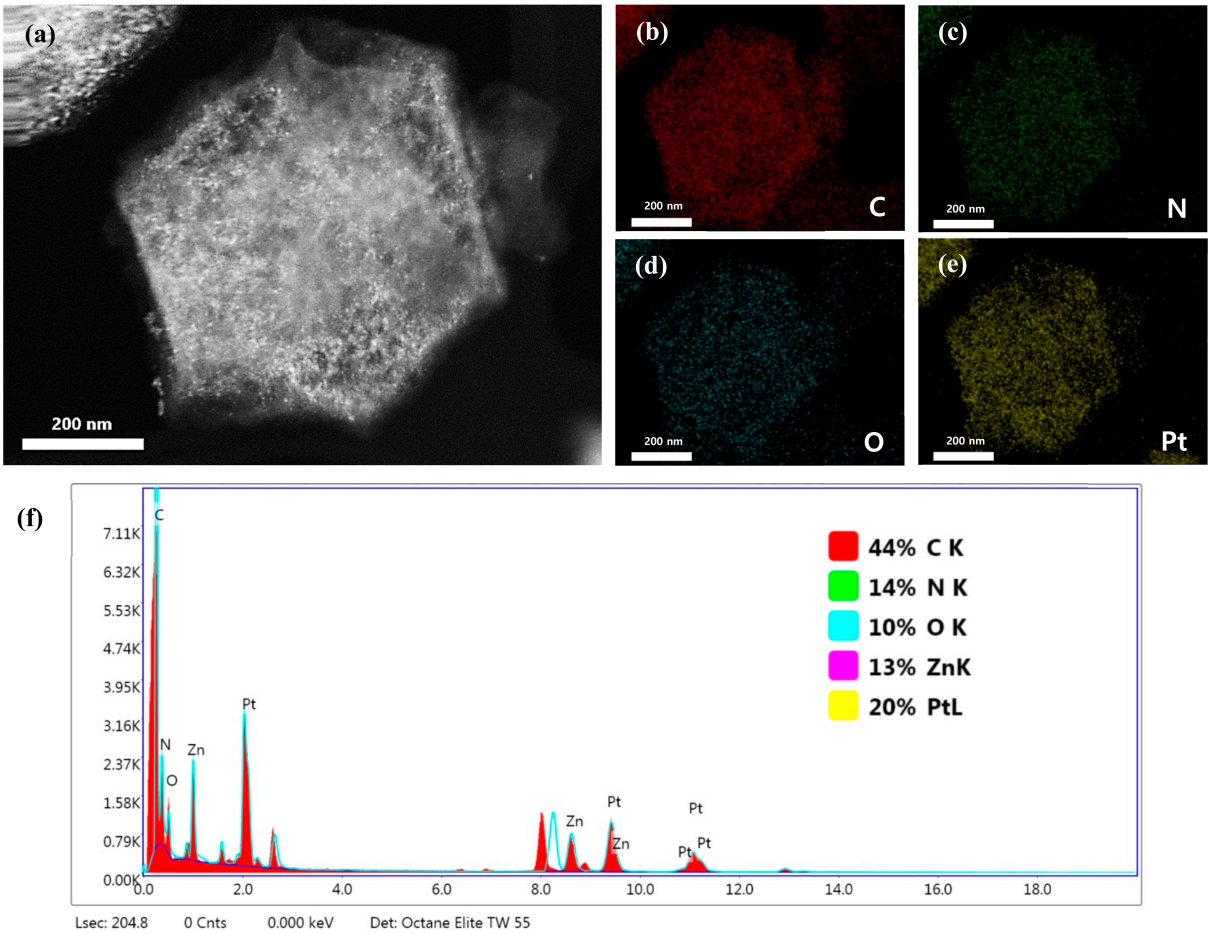

Figure S9. STEM images of (a) Pt/Meso-ZIF-NC-6, (b-e) Elemental mapping image, and (f) The sum spectrum of elemental mapping analysis for Pt/Meso-ZIF-NC-6.

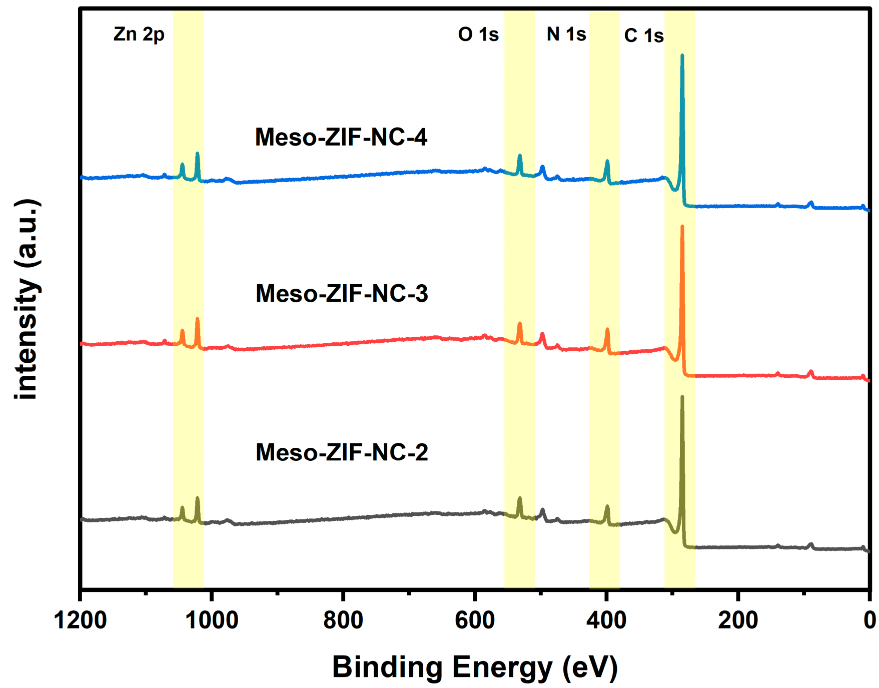

Figure S10. XPS patterns of Meso-ZIF-NC-2,3,4.

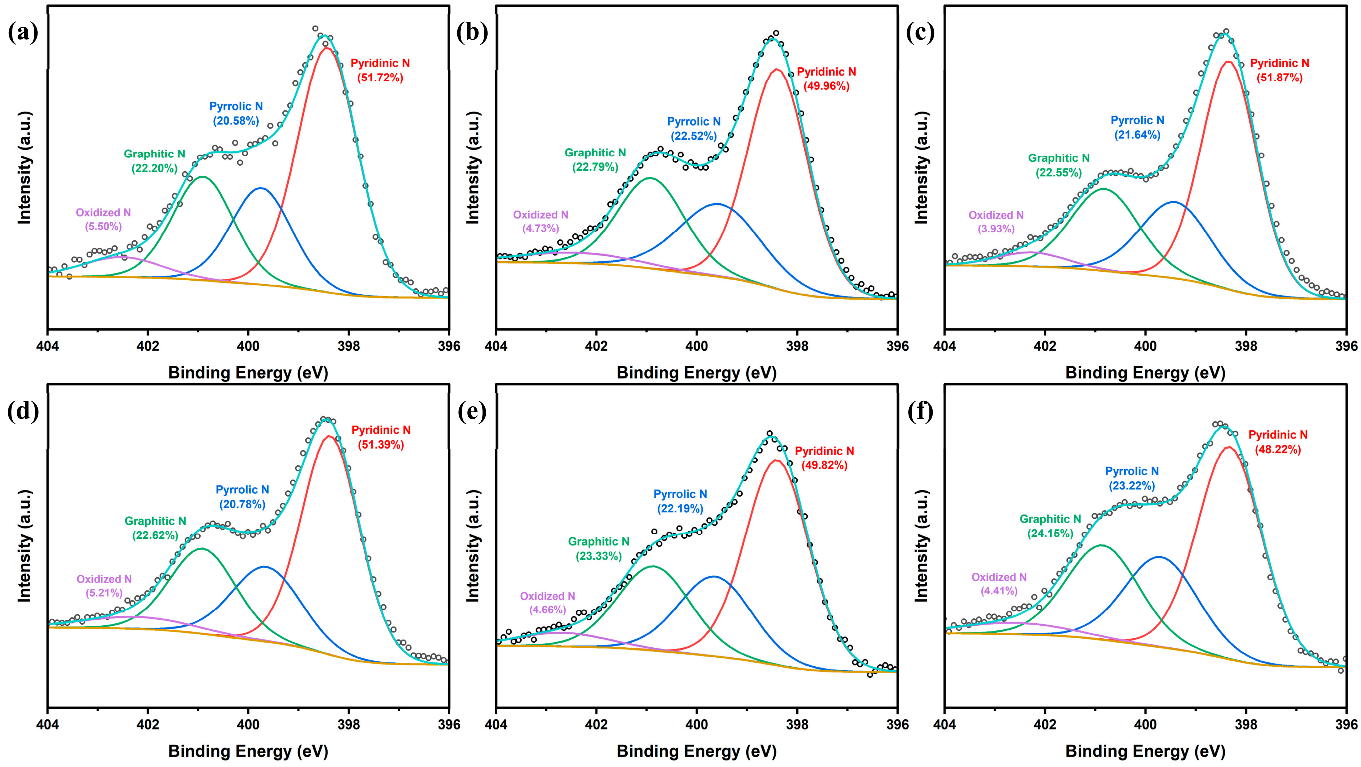

Figure S11. XPS N 1s spectra of Meso-ZIF-NC samples: (a) Meso-ZIF-NC-1, (b) Meso-ZIFNC-2, (c) Meso-ZIF-NC-3, (d) Meso-ZIF-NC-4, (e) Meso-ZIF-NC-5, and (f) Meso-ZIF-NC-6.

Table S1. XPS atomic percentage analysis of Meso-ZIF-NC-2, 3, 4 based on the survey spectra.

Sample	C (at%)	N (at%)	O (at%)	Zn (at%)	Pt (at%)
Meso-ZIF-NC-2	81.46	10.39	6.48	1.67	-
Meso-ZIF-NC-3	79.78	11.65	6.49	2.05	-
Meso-ZIF-NC-4	81.4	10.96	5.9	1.72	-

Table S2. XPS atomic percentage analysis of Pt/Meso-ZIF-NC-1, 5, 6 based on the survey spectra.

Sample	C (at%)	N (at%)	O (at%)	Zn (at%)	Pt (at%)
Pt/Meso-ZIF-NC-1	75.49	7.32	10.23	2.5	4.47
Pt/Meso-ZIF-NC-5	76.26	10.91	7.2	2.17	3.46
Pt/Meso-ZIF-NC-6	81.3	9.49	6.74	1.26	1.22
